# Surgical caseload and annual volume influence cartilage treatment strategies in primary anterior cruciate ligament reconstruction

**DOI:** 10.1002/ksa.12776

**Published:** 2025-07-07

**Authors:** Dzan Rizvanovic, Markus Waldén, Magnus Forssblad, Anders Stålman

**Affiliations:** ^1^ Department of Molecular Medicine and Surgery Stockholm Sports Trauma Research Center, Karolinska Institutet Stockholm Sweden; ^2^ Capio Artro Clinic, Sophiahemmet Stockholm Sweden; ^3^ Unit of Public Health, Department of Health, Medicine and Caring Sciences Linköping University Linköping Sweden; ^4^ Capio Ortho Center Skåne Malmö Sweden; ^5^ Ortopedi Stockholm Stockholm Sweden

**Keywords:** ACL reconstruction, chondral, clinic, experience, surgery, surgeon

## Abstract

**Purpose:**

To evaluate how surgeon and clinic volume, along with patient‐, injury‐ and surgery‐related factors, influence cartilage injury management in primary anterior cruciate ligament reconstruction (ACLR).

**Methods:**

This retrospective cohort study analysed cartilage treatment (debridement, microfracture, other methods or left in situ) in patients undergoing primary ACLR (2008–2022) using data from the Swedish Knee Ligament Registry. Surgeons and clinics were categorised by registry caseload (low [LC]: <50 ACLRs/surgeon, <500/clinic; high [HC]: ≥50, ≥500) and annual volume (low [LV]: <29 ACLRs/year/surgeon, <56/year/clinic; high [HV]: ≥29, ≥56), yielding four groups: LCLV, LCHV, HCLV, and HCHV. Factors influencing cartilage treatment were assessed using adjusted multivariable logistic regression.

**Results:**

More than one in four patients (11,729, 26.4%) had cartilage injuries at the time of primary ACLR; 17.9% underwent debridement, 7.1% microfracture and 1.4% other treatments. A higher proportion of HCHV surgeons had performed debridement (81.2% vs. 48.8%–64.3%), microfracture (78.6% vs. 24.4%–51.0%) and other methods (32.5% vs. 3.8%–13.4%) during ACLR compared to all other surgeon groups (all *p* < 0.001). HCHV clinics were more likely to treat cartilage injuries, leaving fewer in situ (79.2% vs. 80.3%–85.2%, *p* < 0.001) Adjusted logistic regression analyses showed that LCLV/LCHV clinics had 43.4%–68.5% higher odds of performing microfracture, and that LCHV/HCLV clinics had 41.3%–48.1% lower odds of performing debridement compared to HCHV clinics, all *p* ≤ 0.023. Microfracture and debridement odds increased with ICRS 3–4 lesions and decreased with non‐medial femoral condyle injuries and delayed surgery. Age > 30 years and recent surgery year increased debridement odds, while lesion size ≥ 2 cm² lowered microfracture odds.

**Conclusion:**

Surgeons with the highest caseload and volume performed the broadest range of cartilage treatment techniques, yet treatment strategies appeared driven more by clinic caseload and volume. Cartilage management was also influenced by injury location, size, depth, patient age, time to surgery and year of surgery.

**Level of Evidence:**

Level III.

**Abbreviations**
ACI: autologous chondrocyte implantation
ACL: anterior cruciate ligament
ACLR: anterior cruciate ligament reconstruction
BMI: body mass index
CI: confidence interval
HC: high caseload
HCHV: high caseload high volume
HCLV: high caseload low volume
HT: hamstring tendon
HV: high volume
ICRS: International Cartilage Regeneration & Joint Preservation Society
LC: low caseload
LCHV: low caseload high volume
LCL: lateral collateral ligament
LCLV: low caseload low volume
LFC: lateral femoral condyle
LTP: lateral tibial plateau
LV: low volume
MCL: medial collateral ligament
MFC: medial femoral condyle
MTP: medial tibial plateau
OAT: osteochondral autograft transplantation
OCA: osteochondral allograft
OR: odds ratio
PCL: posterior cruciate ligament
PLC: posterolateral complex
PT: patellar tendon
QT: quadriceps tendon
Ref: reference
SKLR: Swedish Knee Ligament Registry

## INTRODUCTION

Cartilage injuries are commonly encountered during primary anterior cruciate ligament reconstruction (ACLR), with proportions ranging from 16% to 46% [3]. These lesions can impact knee function negatively, making their management a critical yet controversial component of ACLR [8, 26, 28]. The use of various cartilage procedures has grown in popularity [12]. While techniques such as debridement, microfracture, osteochondral autograft transplantation (OAT), osteochondral allograft (OCA), autologous chondrocyte implantation (ACI) and others have been described and utilised, their use and reported outcomes vary, particularly in the setting of combined ACLR and cartilage treatment [5, 14].

Debridement of focal cartilage lesions has been shown to improve subjective knee function in the short term [1, 35]. However, concomitant injuries and procedures, such as ACLR or meniscectomy, may compromise the outcomes of debridement [30, 35]. Conflicting evidence exists regarding the effectiveness of microfracture during ACLR, with studies reporting both favourable and unfavourable postoperative outcomes [14, 23, 24, 30]. Although restorative procedures such as OAT and ACI might offer superior results compared to microfracture, they are infrequently used during primary ACLR, require a considerably longer rehabilitation period, and the supporting literature remains limited [14, 37].

The treatment of cartilage injuries remains complex, and current evidence does not support a definitive recommendation for a single 'one‐fits‐all' approach [5, 8]. Surgical volume is one factor that may influence both treatment decisions and outcomes. In the context of ACLR, higher surgical volume has been associated with the greater use of diverse techniques, improved tunnel placement, shorter operative times, enhanced patient‐reported outcomes, reduced readmission rates, lower costs and higher rates of meniscal repair [16, 19, 20, 27, 28, 29, 31]. However, the influence of surgical volume on the specific management of cartilage injuries during ACLR has not been previously investigated. This study aimed to evaluate the impact of surgical volume, along with patient, injury‐ and surgery‐related factors, on the management of cartilage injuries in primary ACLR. We hypothesised that surgical volume would influence cartilage management and that surgeons and clinics with lower caseloads and volumes would be less likely to treat cartilage injuries during primary ACLR.

## METHODS

### Study design and patient selection

Data from the Swedish Knee Ligament Registry (SKLR) were analysed in this retrospective cohort study. The SKLR was established in 2005 as a nationwide registry, capturing over 90% of all ACLRs performed annually [18]. Surgeons report information on injury and surgical characteristics, such as injury cause, time from injury to surgery, the presence of concomitant injuries, surgical duration and all performed surgical procedures in a standardised web‐based protocol. Any concomitant cartilage injuries with the ACLR were documented with details on their location, size, and grade according to the International Cartilage Regeneration & Joint Preservation Society (ICRS) [2]. Additionally, the treatment approach was recorded, including debridement, microfracture, other methods (OAT, ACI or unspecified techniques), or leaving the injury in situ. This study was approved by the Regional Ethical Review Board in Stockholm (2016/1613‐31/2).

Patients who underwent primary index ACLR between 1 January 2008 and 31 December 2022, were screened for eligibility. Patients were included if a cartilage injury was documented at the time of ACLR. Exclusion criteria were as follows: patients younger than 16 years, those with multiple concomitant injuries (such as posterior cruciate ligament injury, other ligament injuries requiring treatment, fractures or tendon/vascular/nerve injuries), those who received grafts other than hamstring, patellar or quadriceps tendon autografts, and patients with missing surgeon codes.

### Surgical volume

Surgeons and clinics were classified based on their overall experience and recent surgical activity, using two parameters: the total number of ACLRs performed before the index procedure (total registry caseload) and the number of ACLRs performed in the year of surgery (annual volume). Based on these factors, surgeons and clinics were categorised into four groups: low caseload and low annual volume (LCLV), low caseload and high annual volume (LCHV), high caseload and low annual volume (HCLV), and high caseload and high annual volume (HCHV), respectively. The thresholds for high and low volume were determined according to prior research from the SKLR [28]. Surgeons with fewer than 50 ACLRs in total and performing fewer than 29 ACLRs per year were classified as LCLV. Clinics were also categorised as LCLV if they had performed fewer than 500 ACLRs in total and had an annual volume below 56 ACLRs per year. Those exceeding these cut‐offs were categorised as HCHV. During the study period, surgeons and clinics could be classified as low‐ or high‐caseload or volume depending on when the primary ACLR was performed.

### Outcome

The primary outcome was the choice of cartilage management strategy (debridement, microfracture, other methods, or left in situ) during primary ACLR, analysed in relation to surgical volume and patient‐ and injury‐specific factors.

### Statistical analysis

SPSS Statistics Version 29 (IBM Corp) was used for all statistical analyses. Standard descriptive statistics summarised all variables. The median (25th–75th percentiles) was reported for non‐normally distributed continuous variables, with the Kruskal–Wallis test used for between‐group comparisons. Categorical variables were presented as counts and percentages, and the *χ*² test was applied for between‐group comparisons.

To identify factors influencing overall cartilage treatment, including debridement and microfracture specifically, in primary ACLR, we performed binary multivariable logistic regression analyses. Adjustments were made for patient age, sex, activity at the time of injury (pivoting sports: yes/no), meniscal injury, time from injury to surgery, year of surgery, cartilage injury characteristics (location, area, and ICRS grade), and surgeon and clinic volume groups. Age (≤20, 21–30, 31–40, and >40 years) and time from injury to surgery (<3, 3–6, 6–12, 12–24, and ≥24 months) were categorised to facilitate result interpretation. The year of surgery (2008–2022) was included to account for time trends. Body mass index (BMI) was excluded due to missing data in nearly half of the cases. To study factors influencing treatment decisions, only patients with one registered focal cartilage injury were included in the regression analyses, as they were eligible for a single treatment option. Results are reported as odds ratios (OR) with 95% confidence intervals (CIs). Statistical significance was set at *p* < 0.05.

## RESULTS

**Figure 1:**
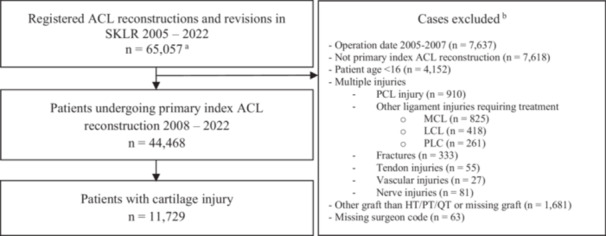
Flowchart of patient selection. ACL, anterior cruciate ligament; HT, hamstring tendon; LCL, lateral collateral ligament; MCL, medial collateral ligament; PCL, posterior cruciate ligament; PLC, posterolateral complex; PT, patellar tendon; QT, quadriceps tendon; SKLR, Swedish Knee Ligament Registry. ^a^ *n* patients = 58,878. ^b^Patients may appear in several groups.

Of 44,468 patients, 11,729 (26.4%) had cartilage injuries during primary ACLR (Figure 1). These were operated on by 284 surgeons at 89 clinics across Sweden.

### Patient, injury and treatment characteristics by surgical volume

Patient demographics, including injury and treatment characteristics, by surgeon and clinic groups are presented in Table 1. Patients operated on by HCHV surgeons had more severe cartilage injuries, with higher proportions of injuries measuring ≥2 cm², full‐thickness ICRS grade 3–4 injuries, and multifocal injuries compared to those treated by other surgeons (all *p* < 0.001). Similar patterns to those seen among surgeons were also observed at the clinic level, with HCHV clinics treating more severe cartilage injuries (all *p* < 0.001).

**Table 1:** Patient, injury and treatment characteristics by surgeon and clinic groups.

	Surgeon group					Clinic group				
	LCLV	LCHV	HCLV	HCHV	*p* value	LCLV	LCHV	HCLV	HCHV	*p* value
Patients, *n*	1317 (28)	249 (18)	2609 (27)	7554 (26)	<0.001	2919 (29)	2542 (29)	559 (24)	5709 (25)	<0.001
Age at surgery, year	30 (22–40)	33 (25–42)	30 (22–41)	31 (23–42)	<0.001	31 (22–41)	31 (22–41)	30 (23–41)	31 (23–42)	0.058
Sex					0.029					0.010
Male	842 (64)	157 (63)	1575 (60)	4512 (60)		1795 (62)	1571 (62)	356 (64)	3364 (59)	
Female	475 (36)	92 (37)	1034 (40)	3042 (40)		1124 (39)	971 (38)	203 (36)	2345 (41)	
BMI, kg/m^2^	25 (23–28)	26 (24–28)	25 (23–28)	25 (23–27)	0.005	25 (23–28)	25 (23–27)	26 (24–28)	24.9 (23–27)	<0.001
Pivoting sport injury	714 (54)	127 (51)	1334 (51)	3782 (50)	0.048	1513 (52)	1356 (53)	283 (51)	2805 (49)	0.003
Meniscal tear	790 (60)	150 (60)	1538 (59)	4685 (62)	0.036	1693 (58)	1491 (59)	341 (61)	3638 (64)	<0.001
Time to surgery, mo	12 (7–26)	10 (5–22)	11 (6–29)	9 (5–23)	<0.001	11 (6–27)	10 (5–23)	13 (6–32)	9 (5–24)	<0.001
Operating time, min	95 (74–119)	90 (74–110)	80 (65–90)	67 (55–82)	<0.001	80 (65–100)	70 (55–90)	80 (65–105)	70 (55–85)	<0.001
Injury characteristics										
Location^a^										
Patella	263 (20)	51 (21)	502 (19)	1684 (22)	0.006	553 (19)	617 (24)	103 (18)	1227 (22)	<0.001
Trochlea	104 (8)	29 (12)	237 (9)	1077 (14)	<0.001	207 (7)	328 (13)	44 (78)	868 (15.)	<0.001
MFC	892 (68)	168 (68)	1739 (67)	5207 (69)	0.178	1975 (68)	1706 (67)	393 (70)	3932 (69)	0.251
LFC	283 (22)	44 (18)	500 (19)	1688 (22)	0.003	522 (18)	594 (23)	121 (22)	1278 (22)	<0.001
MTP	281 (21)	41 (17)	508 (20)	1459 (19)	0.217	538 (18)	538 (21)	99 (18)	1114 (20)	0.052
LTP	249 (19)	49 (20)	525 (20)	2042 (27)	<0.001	547 (19)	629 (25)	71 (13)	1618 (28)	<0.001
Focal lesion	855 (65)	168 (68)	1735 (67)	4703 (62)	<0.001	1998 (69)	1578 (62)	370 (66)	3515 (62)	<0.001
Area ≥ 2 cm^2^	339 (26)	57 (23)	770 (30)	3416 (45)	<0.001	856 (29)	1080 (43)	165 (30)	2481 (44)	<0.001
ICRS grade 3–4	235 (18)	54 (22)	559 (21)	1935 (26)	<0.001	603 (21)	546 (22)	106 (19)	1528 (27)	<0.001
Treatment^a^										
Debridement	231 (18)	52 (21)	436 (17)	1385 (18)	0.166	525 (18)	332 (13)	78 (14)	1169 (21)	<0.001
Microfracture	74 (6)	23 (9)	165 (6)	576 (8)	0.009	190 (7)	202 (8)	32 (6)	414 (7)	0.110
Other method	16 (1)	2 (1)	32 (1)	112 (2)	0.592	33 (1)	51 (2)	6 (1)	72 (1)	0.022
Left in situ	1076 (82)	185 (74)	2133 (82)	6107 (81)	0.032	2345 (80)	2,161 (85)	476 (85)	4519 (79)	<0.001

*Note*: Data are reported as median (25th–75th percentile) or number (percentage). Missing patient values: BMI *n* = 5380; time to surgery *n* = 1327; operating time *n* = 540.

Abbreviations: BMI, body mass index; HCHV, high caseload and high volume; HCLV, high caseload and low volume; ICRS, International Cartilage Regeneration & Joint Preservation Society; LCHV, low caseload and high volume; LCLV, low caseload and low volume; LFC, lateral femoral condyle; LTP, lateral tibial plateau; MFC, medial femoral condyle; MTP, medial tibial plateau.

^a^ Patients may have lesions at several locations and receive more than one treatment option.

A significantly higher proportion of patients operated on by LCHV surgeons underwent microfracture (9.2%) compared to those undergoing ACLR by other surgeon groups (5.6%–7.6%; *p* = 0.009). LCHV surgeons also had the lowest proportion of cartilage injuries left in situ (74.3% vs. 80.8%–81.8%, *p* = 0.032). Among the clinics, HCHV ones performed debridement more often (20.5%) and showed the lowest proportion of leaving the cartilage injury in situ (79.2%) compared to other clinic groups (debridement: 13.1%–18.0%, *p* < 0.001, left in situ: 80.3%–85.2%, *p* < 0.001). Additionally, HCHV surgeons and clinics reported higher proportions of concomitant meniscal tears, shorter times to surgery, and reduced operating times compared to other surgical volume groups (all *p* ≤ 0.036).

### Cartilage treatment strategies

**Figure 2:**
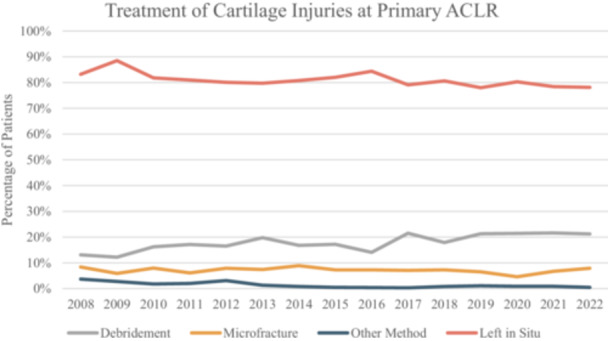
Percentage of patients receiving various cartilage treatments over time during primary ACLR. Patients with several cartilage lesions may receive more than one treatment option. ACLR, anterior cruciate ligament reconstruction.

The distribution of cartilage treatments received by patients over time is presented in Figure 2. The choice of treatment remained unchanged throughout the study period. More than four out of five (81.0%) patients had at least one cartilage injury that was left in situ during primary ACLR. Debridement was the most common treatment (17.9%), followed by microfracture (7.1%) and other methods (1.4%). Among patients with a focal ICRS grade 3–4 injury (12.4%), 64.2% received cartilage treatment, compared to 18.1% of those with a focal ICRS grade 1–2 injury (51.3%).

### Cartilage treatment strategies by surgical volume

**Figure 3:**
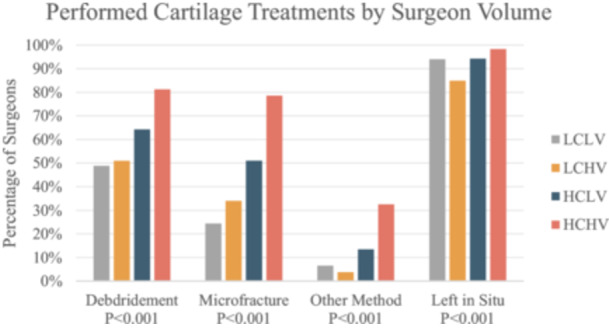
Percentage of surgeons, categorised by volume group, performing at least one cartilage treatment at the time of primary ACLR. The number of surgeons per volume group were as follows: LCLV, 201; LCHV, 53; HCLV, 157; and HCHV, 117. Note that each surgeon may appear in one or several volume groups. HCHV, high caseload and high volume; HCLV, high caseload and low volume; LCHV, low caseload and high volume; LCLV, low caseload and low volume.

The majority of surgeons (70.8%) were categorised as LCLV, while 41.2% achieved HCHV at some point in their careers. Among all surgeons, 193 (68.0%) performed at least one cartilage debridement during primary ACLR, 159 (55.9%) conducted microfracture, 66 (23.2%) performed other treatments, and 275 (96.8%) had at some point left the cartilage injury in situ. The proportion of performed cartilage treatments among surgeon volume groups is demonstrated in Figure 3. A higher proportion of HCHV surgeons had performed debridement (81.2% vs. 48.8%–64.3%), microfracture (78.6% vs. 24.4%–51.0%), and other methods (32.5% vs. 3.8%–13.4%) during ACLR compared to all other surgeon groups, all *p* < 0.001.

**Figure 4:**
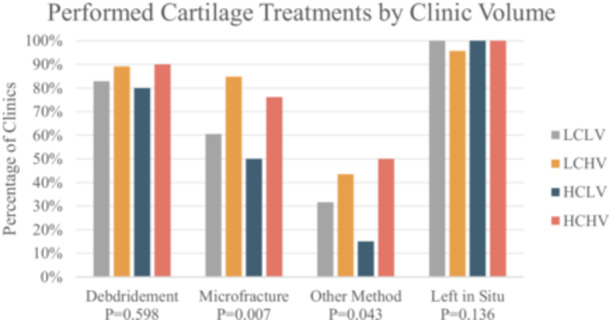
Percentage of clinics, categorised by volume group, performing at least one cartilage treatment at the time of primary ACLR. The number of clinics per volume group were as follows: LCLV, 76; LCHV, 46; HCLV, 20; and HCHV, 30. Note that each clinic may appear in one or several volume groups. HCHV, high caseload and high volume; HCLV, high caseload and low volume; LCHV, low caseload and high volume; LCLV, low caseload and low volume.

Regarding the clinics, 85.4% performed ACLRs as LCLV, whereas 33.7% reached the volumes of HCHV. Additionally, 82 clinics (92.1%) reported at least one cartilage debridement at the time of primary ACLR, 70 (78.7%) had performed microfracture, 45 (50.6%) conducted other treatments, and 89 (100.0%) had left cartilage injuries in situ. The proportion of registered cartilage treatments among clinic volume groups is presented in Figure 4. A greater proportion of clinics with higher annual volumes (LCHV and HCHV) had conducted microfracture and other treatment methods compared to those with lower annual volumes (*p* = 0.07 and *p* = 0.043, respectively).

### Factors influencing the odds of cartilage treatment

Factors influencing overall cartilage treatment, as well as debridement and microfracture specifically, among patients with focal injuries are presented in Table 2. There were 6560 patients included in each model. Of these, 1785 received cartilage treatment: 1211 underwent debridement, 519 were treated with microfracture and 55 with other methods.

**Table 2:** Results of adjusted logistic regression analyses of factors influencing treatment strategies of focal cartilage injuries during primary ACLR.

	Treatment (*n* = 6560)		Debridement (*n* = 6560)		Microfracture (*n* = 6560)	
Variable	OR (95% CI)	*p*‐Value	OR (95% CI)	*p* value	OR (95% CI)	*p* value
Age at surgery, year						
≤20	Ref		Ref		Ref	
21–30	1.278 (1.069–1.526)	0.007	1.192 (0.985–1.444)	0.072	1.296 (0.921–1.822)	0.136
31–40	1.586 (1.309–1.923)	<0.001	1.581 (1.290–1.938)	<0.001	1.208 (0.838–1.739)	0.311
>40	1.500 (1.239–1.817)	<0.001	1.621 (1.326–1.980)	<0.001	0.879 (0.607–1.272)	0.493
Sex						
Male	Ref		Ref		Ref	
Female	0.933 (0.821–1.060)	0.288	0.927 (0.810–1.060)	0.268	0.994 (0.781–1.266)	0.964
Time to surgery, month						
<3	Ref		Ref		Ref	
3–6	0.941 (0.765–1.158)	0.567	1.172 (0.941–1.459)	0.156	0.576 (0.395–0.838)	0.004
6–12	0.899 (0.736–1.097)	0.294	1.171 (0.948–1.446)	0.143	0.489 (0.339–0.705)	<0.001
12–24	0.783 (0.629–0.976)	0.030	0.935 (0.738–1.184)	0.577	0.602 (0.406–0.891)	0.011
≥24	0.671 (0.544–0.826)	<0.001	0.771 (0.616–0.967)	0.024	0.612 (0.430–0.872)	0.007
Year of surgery	1.032 (1.016–1.048)	<0.001	1.044 (1.027–1.061)	<0.001	—	
Location						
MFC	Ref		Ref		Ref	
Patella	0.918 (0.743–1.135)	0.429	0.986 (0.793–1.224)	0.896	0.507 (0.224–1.151)	0.104
Trochlea	0.511 (0.368–0.709)	<0.001	0.582 (0.404–0.838)	0.004	0.723 (0.462–1.130)	0.155
LFC	0.686 (0.558–0.842)	<0.001	0.593 (0.468–0.751)	<0.001	1.142 (0.820–1.592)	0.432
MTP	0.468 (0.331–0.660)	<0.001	0.552 (0.383–0.797)	0.002	0.281 (0.130–0.607)	0.001
LTP	0.363 (0.283–0.467)	<0.001	0.486 (0.377–0.626)	<0.001	0.146 (0.070–0.305)	<0.001
Area ≥2 cm^2^	—		—		0.712 (0.562–0.902)	0.005
ICRS grade 3–4	8.017 (6.946‐9.252)	<0.001	1.474 (1.262‐1.722)	<0.001	72.684 (52.725–100.200)	<0.001
Surgeon volume						
LCLV	1.197 (0.973–1.472)	0.089	—		1.004 (0.662–1.522)	0.987
LCHV	1.503 (1.021–2.212)	0.039	—		2.732 (1.379–5.415)	0.004
HCLV	0.900 (0.765–1.060)	0.207	—		0.873 (0.643–1.186)	0.386
HCHV	Ref		Ref		Ref	
Clinic volume						
LCLV	1.000 (0.849–1.177)	0.999	0.917 (0.786–1.069)	0.267	1.434 (1.050–1.957)	0.023
LCHV	0.738 (0.620–0.878)	<0.001	0.587 (0.484–0.713)	<0.001	1.685 (1.260–2.253)	<0.001
HCLV	0.560 (0.410–0.765)	<0.001	0.519 (0.373–0.723)	<0.001	0.900 (0.495–1.634)	0.729
HCHV	Ref		Ref		Ref	

*Note*: Data are reported as odds ratios (95% CIs). All values are adjusted for the included variables in each model, and for activity at time of injury and presence of meniscal tears. Treatment was defined as receiving either debridement, microfracture or another method.

Abbreviations: ACLR, anterior cruciate ligament reconstruction; CI, confidence interval; HCHV, high caseload and high volume; HCLV, high caseload and low volume; ICRS, International Cartilage Regeneration & Joint Preservation Society; LCHV, low caseload and high volume; LCLV, low caseload and low volume; LFC, lateral femoral condyle; LTP, lateral tibial plateau; MFC, medial femoral condyle; MTP, medial tibial plateau; OR, odds ratio; Ref, reference.

Surgical volume influenced the treatment of cartilage injuries during ACLR. LCHV surgeons had significantly increased odds for any cartilage treatment and for microfracture compared to HCHV surgeons (*p* = 0.039 and *p* = 0.004, respectively). Similarly, clinics with low total caseloads (LCLV and LCHV) had increased odds for microfracture compared to HCHV clinics (*p* = 0.023 and *p* < 0.001, respectively). In contrast, LCHV and HCLV clinics had decreased odds for any cartilage treatment and for debridement compared to HCHV clinics (all *p* < 0.001). No significant differences in overall cartilage treatment were observed between LCLV and HCHV surgeons and clinics.

The odds of receiving any cartilage treatment increased significantly with patient age (all *p* ≤ 0.007) and for ACLRs performed in more recent years (*p* < 0.001). Conversely, ACLRs delayed more than 1 year from injury significantly decreased the odds of cartilage treatment (all *p* ≤ 0.03). For debridement, this decrease was significant after 2 years (*p* = 0.024), while for microfracture, there was a significant decrease after three months (all *p* ≤ 0.011).

The characteristics of the cartilage injury also influenced the choice of treatment. Injuries located outside the medial femoral condyle (MFC) decreased the odds of receiving cartilage treatment and debridement (all *p* ≤ 0.01, except for patellar injuries, which were non‐significant). Conversely, full‐thickness ICRS grade 3–4 injuries increased the odds of all treatment options, especially for microfracture (all *p* < 0.001). The size of the cartilage injury influenced only the odds of microfracture, with larger injuries reducing the odds ratio (*p* < 0.001).

## DISCUSSION

The main finding of this study was that approximately one in four patients presented with cartilage injuries at primary ACLR, and the management of these injuries varied by surgical volume. HCHV surgeons performed a wider range of cartilage procedures, while treatment strategies appeared to be driven by clinic caseload and volume, with more debridement and less microfracture performed at HCHV clinics. Management was also influenced by location, size and depth of injury, patient age, time to surgery, and year of surgery. These findings support our hypothesis that surgical volume impacts cartilage management and align with previous research demonstrating a volume‐related effect on ACLR techniques, particularly regarding graft selection and meniscal injury treatment [27, 29].

Among the various cartilage treatment strategies, debridement emerged as the most commonly performed procedure. Debridement does not appear to negatively affect patient‐reported outcomes for full‐thickness (ICRS 3–4) lesions [34], which could explain its frequent use and slight increase over time. Moreover, HCHV clinics demonstrated increased odds of performing debridement, suggesting that it is preferred as a straightforward intervention to avoid the potential drawbacks of microfracture or more time‐consuming and costly alternatives when these are not feasible. However, no differences were observed between clinics with the lowest and highest caseloads and volumes. This similarity may suggest that debridement is a widely accepted standard of care or reflect shared clinical practices or guidelines that override volume‐related variation. Although nearly one‐third of surgeons had never performed debridement, surgeon volume had no significant impact on debridement odds, suggesting that clinic‐level factors play a greater role in debridement decisions than individual surgeon experience.

Microfracture has long been a first‐line cartilage repair technique, improving short‐ to mid‐term knee function, particularly in small lesions and among younger patients [11, 23]. However, its durability is questionable, with outcomes deteriorating as early as 2 years postoperatively [11, 13, 30]. Microfracture has also been linked to worse outcomes than restorative techniques, such as ACI and OAT, and to higher failure rates in subsequent cartilage restoration procedures [14, 22, 32, 37].

Despite these concerns, no declining trend in microfracture utilisation was observed, and no significant differences in the odds of performing microfracture during ACLR were found between surgeons in the lowest and highest volume groups. This lack of difference aligns with an international surgeon survey on cartilage lesion management, showing no association between surgeon experience and microfracture treatment [21]. Although we found an increased preference for microfracture among early‐career surgeons with high annual volumes, possibly indicating a greater inclination to intervene, these results should be interpreted with caution, as only 2.1% of patients underwent ACLR by LCHV surgeons. In contrast, recently trained orthopaedic surgeons in the United States have shown a decline in microfracture use [9], a discrepancy that may reflect regional practice variations [21]. Importantly, LCLV and LCHV clinics had 43%–69% higher odds of performing microfracture. This may suggest that clinics with high caseload and volume are more selective in identifying suitable patients for the procedure.

Although variability in indications, operative techniques, and rehabilitation protocols for patients undergoing microfracture has been reported, there is general consensus that smaller lesion size and, especially, higher ICRS grades are key factors in treatment selection [6, 11, 21]. In line with this, our study found that lesions ≥2 cm² had 29% lower odds of microfracture, while those classified as ICRS grade 3–4 had 73‐fold higher odds of receiving microfracture treatment, reinforcing the importance of lesion severity in surgical decision‐making. ICRS grade 3–4 also increased debridement odds by 47%, aligning with previous research [17]. However, the variation in treatment decisions among centres of the same volume classification, where the same lesion might be managed with microfracture at one center and debridement at another, remains unexplained and warrants further investigation. Injury location also influenced treatment. Compared to the MFC, tibial‐sided lesions had lower odds of both debridement and microfracture, consistent with prior studies [10, 17, 21].

Interestingly, microfracture odds declined after three months from injury to surgery, while debridement became less common after 2 years. This may suggest that acute cartilage injuries suitable for microfracture are prioritised for earlier intervention, while debridement is more commonly performed in chronic cases. Moreover, patients undergoing ACLR after two years may have less symptomatic cartilage injuries that do not require treatment. Prior research has shown an increased prevalence of cartilage damage and degeneration with ACL injury chronicity and advancing patient age, both of which were accounted for in the analyses [4, 10, 33]. Therefore, the higher proportion of cartilage injuries in LCLV settings may be attributed to longer times from injury to ACLR. Patient age also influenced cartilage treatment, with odds increasing by 28% in those aged 21–30 years, 59% in those aged 31–40 years, and 50% in patients over 40 years. This may seem counterintuitive, as older age has been considered a prognostic factor for poorer outcomes following cartilage surgery [25, 36], though this has been questioned recently [7]. In this cohort, most cartilage lesions were treated with debridement or microfracture, consistent with another registry study showing these procedures are more common in older patients [10].

Cartilage restoration procedures have gained popularity and are considered safe to perform alongside other interventions [12]. Various restoration techniques exist, each with its own advantages and limitations, and they differ widely in their indications [8, 15]. While these procedures may offer superior outcomes compared to treatments such as microfracture, evidence remains scarce, particularly in the setting of ACLR [14, 32, 37]. Only 1.4% of patients with cartilage injuries undergoing primary ACLR were treated with OAT, ACI or another unspecified technique, introducing potential bias due to the small, heterogeneous sample, and preventing meaningful regression analysis. These techniques may be underrepresented, as they might be performed as part of a staged procedure, separate from the ACLR. Consequently, no conclusions can be drawn from these data regarding the impact of surgical volume on restorative treatments during primary ACLR, emphasising the need for further research in this area.

Cartilage injuries have been linked to significantly worse subjective outcomes following ACLR [8, 28]. Despite this, general treatment patterns have remained largely unchanged over the 15‐year study period, reflecting the persistent challenges in optimising cartilage management. In line with this stagnation, most lesions were left untreated in situ, a finding consistent with a systematic review on cartilage management during ACLR [8]. Given the debated benefits and potential risks of surgical cartilage treatment, non‐intervention may be appropriate in many cases. However, HCHV clinics had the lowest proportion of untreated cartilage injuries, suggesting a greater inclination toward active management, even after adjusting for differences in lesion severity. This may indicate that these centres have more established treatment protocols and greater access to specialised expertise.

Ultimately, these findings highlight that surgical volume, particularly at the clinic level, can influence how cartilage injuries are managed during ACLR. Given the high prevalence of such injuries, clinicians and patients should be aware that institutional experience and treatment protocols may affect decision‐making and outcomes.

### Limitations

Beyond the acknowledged limitations already discussed, several additional factors should be considered. The rationale behind treatment decisions remains unclear, as it is unknown whether these injuries were symptomatic or merely incidental findings, and their underlying cause, whether traumatic or degenerative, was not specified. The registry does not capture any cartilage procedures performed prior to the primary ACLR, and such interventions could therefore not be accounted for in the analyses. Moreover, since the regression analyses focused on focal injuries to avoid the complexity of assessing factors influencing treatment allocation among multiple injuries, the findings may not be applicable to patients with multiple cartilage injuries. Lesion severity differences may have introduced selection bias, with severe injuries likely treated more often at HCHV centres. However, patient‐, injury‐, and surgery‐related factors were accounted for to reduce confounding.

Another potential limitation was reporting bias by the operating surgeon. For example, LCLV surgeons reported a lower proportion of injuries to the lateral compartment, suggesting possible differences in injury detection, reporting accuracy, or patient selection. Surgical technique heterogeneity within treatment groups, made it difficult to specify exact approaches across surgeons and clinics. Although accounting for both surgeon and clinic volumes, including their total caseload and annual ACLRs, complicated the interpretation of results, we recommend that future research continue to incorporate these variables to provide deeper insights into how individual expertise and clinic routines shape ACLR outcomes.

## CONCLUSION

Surgeons with the highest caseload and volume performed the broadest range of cartilage treatment techniques, yet treatment strategies appeared to be driven more by clinic caseload and volume. Cartilage management was also influenced by location, size and depth of injury, patient age, time to surgery, and year of surgery.

## AUTHOR CONTRIBUTIONS

All authors contributed to the conception and design of the study. Dzan Rizvanovic was responsible for data extraction and analysis from SKLR. Dzan Rizvanovic wrote the initial draft of the manuscript. Markus Waldén, Magnus Forssblad and Anders Stålman critically revised the manuscript. All authors (Dzan Rizvanovic, Markus Waldén, Magnus Forssblad and Anders Stålman) had full access to the data, participated in interpretation of the findings, and approved the final version. Anders Stålman is the study guarantor.

## CONFLICT OF INTEREST STATEMENT

Anders Stålman is a member of the steering committee of the Swedish Knee Ligament Registry (SKLR). The remaining authors declare no conflicts of interest.

## ETHICS STATEMENT

The study was approved by the Regional Ethical Review Board in Stockholm (2016/1613‐31/2).

## Data Availability

The data supporting the findings of this study are available from the corresponding author upon reasonable request and approval from the Swedish Knee Ligament Registry committee.
